# Insurance companies’ point of view toward moral hazard incentives

**Published:** 2016-11-05

**Authors:** Elahe Khorasani, Mahmoud Keyvanara, Manal Etemadi, Somayeh Asadi, Mahan Mohammadi, Maryam Barati

**Affiliations:** 1 PhD Candidate, Department of Pharmacoeconomics and Pharmaceutical Administration, School of Pharmacy, Tehran University of Medical Sciences, Tehran, Iran; School of Pharmacy, Isfahan University of Medical Sciences, Isfahan, Iran;; 2 Associate Professor, Department of Healthcare Management, Social Determinants of Health Research Center, School of Management and Medical Informatics, Isfahan University of Medical Sciences, Isfahan, Iran;; 3 PhD Candidate, Department of Health Services Management and Health Policy, Health Management and Economics Research Center, Iran University of medical Sciences, Tehran, Iran;; 4 BSc, Department of Healthcare Management, Isfahan University of Medical Sciences, Isfahan, Iran;; 5 MSc, Department of Healthcare Management, Isfahan University of Medical Sciences, Isfahan, Iran.

**Keywords:** *Moral hazard*, *Patients*, *Insurance organizations*

## Abstract

Moral hazards are the result of an expansive range of factors mostly originating in the patients’ roles. The objective of the present study was to investigate patient incentives for moral hazards using the experiences of experts of basic Iranian insurance organizations.

This was a qualitative research. Data were collected through semi-structured interviews. The study population included all experts of basic healthcare insurance agencies in the City of Isfahan, Iran, who were familiar with the topic of moral hazards. A total of 18 individuals were selected through purposive sampling and interviewed and some criteria such as data reliability and stability were considered. The anonymity of the interviewees was preserved. The data were transcribed, categorized, and then, analyzed through thematic analysis method.

Through thematic analysis, 2 main themes and 11 subthemes were extracted. The main themes included economic causes and moral-cultural causes affecting the phenomenon of moral hazards resulted from patients’ roles. Each of these themes has some sub-themes.

False expectations from insurance companies are rooted in the moral and cultural values of individuals. People with the insurance coverage make no sense if using another person insurance identification or requesting physicians for prescribing the medicines. These expectations will lead them to moral hazards. Individuals with any insurance coverage should consider the rights of insurance agencies as third party payers and supportive organizations which disburden them from economical loads in the time of sickness.

## Introduction

Globally, 8% of countries’ gross domestic product (GDP) goes toward healthcare costs (1, 2). In most developing countries, about 5% to 10% of the costs of each government are allocated to the health sector (1, 3). In Iran, about 7% of the GDP is allocated to the costs of the healthcare system (4). A consequence of the rapid growth in healthcare costs is the fact that annually more than 2% of households live below the poverty line. This growth in healthcare costs is because of the increased household demands for healthcare services which can be due to the asymmetric information about the status of individuals’ health (5). 

On the other hand, the need for healthcare and the costs of receiving services are basically related to the uncertainty of those who do not have enough knowledge about their sickness and the treatment costs. Most often, catching a disease is something accidental (6). To provide fair healthcare services, and consequently, support households in the high costs of these services, insurance plans and projects are considered as important policies and coverage plans are established in different countries (7). 

Insurance coverage mostly results in the excessive use of care services by insured individuals. Individuals who are insured pay more attention to direct costs than to the total costs (6). Some healthcare services are only utilized due to the existence of insurance coverage (8). Excessive use means insured individuals purchase inpatient or outpatient services for example, and medicines at the point where the final cost and final benefit are not equal. The cost pooling made individuals to pay less for a service although it motivates them to use services which are not necessary and those that cost more than what they are worth (9-11). These changes in behaviors are due to increases in healthcare costs in a society. These phenomena of changes in behaviors resulting from insurance are defined as moral hazards (12-14). The lack of awareness about the severity or consequences of diseases can result in moral hazards for those who do not have any control over their treatment and their insurance coverage has no limitation in use of healthcare services (15). 

In general, two types of moral hazards can be distinguished; “provider moral hazard” and “consumer moral hazard” (6). Provider moral hazard is explicated in the framework of induced demand. In fact, induced demand refers to referring or selling unnecessary services to clients of the health system and abusing the power of the imbalance between the providers information and the customers information. Different economic and structural factors, behaviors of providers and consumers of services, and the asymmetry of information among them are effective on induced demand which causes use of services and goods with few benefits for the user (customer) (16-18). Consumer moral hazard means that individuals enjoying insurance coverage utilize more care services than those who do not have any insurance because the perceived cost of receiving services is less than the real cost spent for the services (6).

Due to the significance of investigating the causes of moral hazard among patients, the objective of the present study was to investigate patient incentives for moral hazard using the experiences of experts of two basic Iranian insurance agencies which can be summarized into a certain framework for policymakers of the health sector. 

## Method

This qualitative study was conducted in 2014 using in depth interviews. The participants of the research included individuals who were aware of moral hazard aspects. The study population consisted of experts of two Iranian basic healthcare insurance agencies‎ in the city of Isfahan, Iran. The insurance agencies studied included the Iran Health Insurance and the Iranian Social Security Organizations. In addition, insurance experts working in educational hospitals were involved in the study. 

The subjects were selected through purposive sampling method. In other words, those individuals were interviewed who had great knowledge of this field and valuable experiences. The selection of participants continued until reaching data saturation. Accordingly, 18 verbal interviews were conducted. All interviews were recorded, and then, transcribed. The duration of each interview was between 30 and 90 minutes. 

To insure the validity and reliability of interviews, the triangulation technique that includes the use of interview guidelines and the confirmation of the framework of interviews by other researchers was used. The interviews were guided in order to prevent the influence of the researchers’ bias. Frist, some interviews were experimentally conducted before starting the research phases. Then, to approve the validity of the data collected from the interviews, they were analyzed in terms of the accuracy of data collection. To enhance reliability of findings, after extracting the data, the transcriptions of interviews were returned to some of the participants and their comments were applied. Furthermore, the researchers tried to create a theoretical framework with high analytical precision. The conceptual coding method available in interviews contributed to the enrichment of data collection. Criteria such as credibility of the data which is the validity of the study, trustworthiness which is the reliability, and provability which is the capability of final confirmation of the data were considered. The data analysis method was based on the thematic analysis method. 

Stages of data analysis included data extraction, transcription, and storing in the computer and immersion, coding, reflexive remarks, marginal remarks, and memoing. In the first stage after each interview, the text was transcribed, then, immediately typed and stored in the computer. In the next stage, the interview texts were examined and reviewed several times to understand and distinguish data. In the third stage, data were categorized into semantic units (code) in the form of sentences and paragraphs related to the main idea. Semantic units were also reviewed several times. Then, an appropriate code was inscribed for each semantic unit. Accordingly, in each interview, subthemes were separated, and further reduced. Finally, the main themes were recognized. Reflective and marginal signs, ideas, and viewpoints emerging in the researcher’s mind were recorded during the interview and analysis. Research ethical principles were also considered to gain the interviewees’ satisfaction. The participants were informed that interviews were being recorded for the purpose of easy transcription. Anonymity of the interviewees was preserved and the participants were assured of information confidentiality. 

## Results

The ideas of the participants indicated the important role of the patients in creating moral hazards. In the present qualitative research, 2 main themes and 11 subthemes were obtained. The main themes included economic causes and moral-cultural causes affecting moral hazards. 

**Economic causes**

The ideas of participants regarding patients in the domain of economic causes affecting moral hazards are summarized in the subthemes of poverty and economic pressures, the inability to pay for treatment, individuals' inclination to pay the lowest cost, and insurance coverage abuse. 

*Poverty and economic pressures*

Participants presented factors related to patients such as poverty and destitution. [*Poverty, in my point of view, is the main factor*] (Interviewee number 2). [*I think one cause is the destitution of the patients*] (Interviewee number 5). [*Among the insured people, the cause is financial needs*] (Interviewee number 14). [*Regarding the patients, the main cause is poverty. In this case poverty does not mean the ability to pay for household costs, but it means the amount someone wishes to pay for a service with or without insurance*] (Interviewee number 15).

Participants also referred to economic pressures. [*Realities should be stated; this problem occurs in our society due to economic pressures*] (Interviewee number 17). [*However, in a society, with not enough discipline, rapidly increasing inflation, and an imbalanced income-expenses ratio, sure there will be a weakened morality*] (Interviewee number 7). 

*Chronic disease with high medical costs*

Participants mentioned “the inability to pay treatment costs”, “insurance costs”, “the growing rate of out-of-pocket expenses”, “increase in catastrophic costs”, “admission fees”, and “the costs associated with chronic diseases” as factors affecting moral hazards. They also declared that [*when people cannot afford their treatment costs, they will move toward moral hazards*] (Interviewee number 2). [*In that case, they cannot pay for health insurance registration fees, so this may lead to using the insurance identification of others in hospitals*] (Interviewee number 2). 

[*A quick look at the current out-of-pocket expenses rate shows the high probability of catastrophic costs for patients and occurrence of conditions which can destroy their lives or make it very hard to survive. This can be clearly observed in the cases of chemotherapy and special cases*] (Interviewee number 18). 

[*Well, you know, in any hospital admission, those with no insurance coverage face the high costs of treatment, so that would be an incentive to misuse the situation*] (Interviewee number 17). 

[*In the case of patients with chronic diseases, such as those with thalassemia, they know how to abuse the system. For example, they are reimbursed by the health sector, and then, they take the bills to charities or the Red Crescent Society*] (Interviewee number 6).

*Inclination to pay the lowest cost*

A participant pointed to the willingness of most individuals to pay less and use governmental subsides as much as possible. [*For example, insurance coverage is possible by only having an insurance card; so, there will be opportunities to commit crimes to have this advantage and pay less for the services received*] (Interviewee number 4). 

*Insurance coverage abuse*

Some participants considered financial issues and insurance coverage abuse as the causes of moral hazard. [*Greed will cause some people to earn the most out of something” (Interview 5). “I think the most important incentive is to earn money in this way*] (Interviewee number 6).

**Moral-cultural causes**

The participants’ ideas indicated some extents of immoralities in patients’ behaviors; which were presented in the form of the subthemes of false attitudes and expectations of the insured individuals, the decline of spirituality in the society, and cultural poverty and unawareness of the insured.

*False attitudes and expectations of the insured*

Examples of cases of wrong attitudes of the insured and their inappropriate expectations from the insurance organization were presenting unreal excuses to receive more services, considering any money received from insurance companies as a right, seeing the insurance agency as responsible for all treatment costs, receiving all kinds of services after paying insurance premium, receiving the maximum services without requiring them, getting money easily from the insurance organization and compensation without any relation to insurance, pretending to be sick to receive coverage, inadequate understanding about the importance of ID cards of others, and insisting to use the services of special centers with no contracts with the insurance company. [*They ask physicians to prescribe 10 different medicines instead of 4 without needing them, because some drugs receive subsides after being prescribed by a physician*] (Interviewee number 4). 

[*The insured persons consider it their right and claim that they have a legal right, because they pay insurance premium, and the insurance agency should pay all their costs. They have a health insurance card and pay insurance premium monthly; therefore, the cosmetic services that they perform should be paid for by the insurance company, while this is not in the insurance contract; so, this should not be a motivation for misusing the insurance*] (Interviewee number 12). 

[*The definition considered by people for insurance is that they can get money as easily as possible from the insurance and this issue results in many damages or obtaining money due to many damages not under contract to the insurance*] (Interviewee number 7). 

[*There are some cases in which people declare the events in a different way. For example, there has been a conflict, and he/she says that he/she has fallen or the event has occurred during work. It is not the responsibility of insurance agencies ‎to pay for these issues. He/she was working and he/she has an employer who has to pursue the event. They want to present the reality in a different way and misuse the health insurance cards of other people*] (Interviewee number 2). 

[*For example, the patient has had an accident and he has been insured late. He has another person’s health insurance card. When he refers here, it is not important for him that he does not own the health insurance card and says that it belongs to my family, there is no difference in ownership between me and my family*] (Interviewee number 2). [*In some cases an insured person wants to use the services of all insurance centers, for example, hospitals and private institutes, while all centers do not have contracts with insurance agencies*] (Interviewee number 4). 

*The decline of spirituality in the society*

A participant considered the decline of spirituality in the society as a factor in the occurrence of moral hazard and said: [*Spirituality has greatly decreased in the society, and a lot of people have no commitment to this issue morally, then, they claim that the government should provide insurance coverage for them*] (Interviewee number 18). [*Everyone thinks that if they can get some money from insurance agencies, even when it is illegal, they have done a good job and it is religiously legitimate*] (Interviewee number 7). 

*Cultural poverty and unawareness of the insured*

The participants presented cultural poverty and unawareness of the insured individuals as a cause of the occurrence of moral hazard. [*This issue has emerged in our society due to individuals’ unawareness and their lack of high vision*] (Interviewee number 17). [*Now, when somebody, due to his or her unawareness of insurance laws and regulations, thinks that he or she can persuade physicians or authorities of hospitals, as well as insurance experts, by crying, this is cultural poverty*] (Interviewee number 16). 

[*We have a number of patients who act illegally due to their unawareness, due to their cultural level. For example, they think that if the father of a family pays insurance premium, and all of his children, except for one of them, have health insurance cards, he can use the cards of others, or they have cards they have not used for a long time, so the wife believes that because her husband has paid insurance premium and she has not used it, at least her sister should use it*] (Interviewee number 15). 

A participant refers to patients’ unawareness of a good physician and declared that [W*e have problems in the issue of treatment and medicine. People think that if their physicians prescribe ampoules for them, they are proficient, but if they prescribe tablets, they are not*] (Interviewee number 15). [*For example, if I refer to a physician and he prescribes that I should go home and rest up until I am healthy, I will never refer to that physician again. Instead, I will refer to those physicians who prescribe drugs for, say, my runny nose. I claim that these physicians are proficient, because they make me healthy again, but a lot of diseases may not have therapeutic indications*] (Interviewee number 10). 

## Discussion

Participants believed that moral hazards are more likely to occur in patients of lower economic levels. Homaie Rad et al. and Ebrahimnia et al., in their quantitative studies on the medical services of the armed forces indicated that with the decrease in income, moral hazard increases (19, 20). Other similar studies indicated the positive correlation of income with moral hazards (21-23). These results are not consistent with the results of the present study. However, Ashkzari, in his study, indicated that the low income of families is one of the causes of misuse of insurance (24). 

In this study, the unaffordability of treatment costs and coping with cost uncertainty were obtained as the main causes of moral hazards, especially in the case of chronic diseases with repetitive treatment costs, which may lead to misuse of insurance. Koç, in his study, indicated that the rate of moral hazard in physicians who visit patients with chronic diseases is 2 times higher than others (25). These results are not consistent with the present study results. Haddad and Anbaji‎ indicated that the more critical the health status of an individual is, the more likely he/she is to face moral hazards (26). These results are consistent with the results of the present study. 

The participants of this study referred to insurance coverage abuses by some individuals. For instance, they may demand the physician to prescribe more drugs than needed, so they can sell them in the free market and receive extra money as an income source, which is an evident example of abuse of the insurance coverage (27). Ghodoosi et al., in their research on physicians’ insurance frauds, showed that 1% of physicians and 0.8% of specialists, in 2010, committed fraud by drugs and services prescriptions only based on the patients’ demands (28). Amani et al. indicated that 32% of the individuals referring to physicians ask them to prescribe drugs based on their opinion and not the physician’s diagnosis (29). In addition, in 26% of the cases, physicians act according to the patients demands (29). 

Interviewees considered the tendency to pay the lowest costs as another cause of moral hazards. Since individuals under insurance coverage have lower out-of-pocket expenses in case of receiving services, one important origin of moral hazard can be considered as the tendency to pay less. Haddad and Anbaji indicated that %1 increase in out-of-pocket expenses causes a 2.9% decrease in the costs of pharmaceutical and laboratory services (26). 

The participants believed that the individuals’ high expectation to use other people’s insurance cards indicates their attempt to ignore others’ rights in the society. Ashkzari considers low level of commitment to the community as a cause of individuals’ misuse in the form of giving health insurance cards to others for use (24). In fact, the lack of moral commitment to the community and negligence of individuals’ rights are among the causes of moral misuse of the health insurance cards of others. 

The participants identified one of the causes of moral hazards as the tendency of the insured person to use centers which are not under contract with the insurance agency. Meskarpour and Kazemian referred to the tendency of the insured person to refer to health institutions not under contract with insurance agencies (30). This issue reduces the possibility of control and supervision of insurance agencies over insurance costs and the quality of provided care to the insured individuals (30). Turning to a health center with no insurance contract, in which the possibility of insurance experts and supervisors, directly or indirectly, is not possible, increases the probability of moral hazards. Insurance agencies should minimize the possibility of the occurrence of moral hazards in insured individuals in institutions not under contract with insurance agencies through the quantitative and qualitative development of centers under contract with insurance agencies. 

Another issue considered as important by the participants was the decline of spirituality in the society. Religious teachings consider moral deviations as indecent and discourage individuals from committing them. The fading and destruction of moral values in human communities provide the path for the occurrence of moral abnormalities and misuses. Badamchi states that the role of religion in public behaviors has highly faded and only 31% of the subjects in his research believed in the intervention of religion in public behaviors (31). He believes that individuals keep those dimensions of religiosity which do not engender any intervention for their social interactions and overlook the other dimensions (31). 

In the present study, cultural poverty in the society has been considered as the root of many moral hazards in the insured individuals. Visiting many physicians for receiving different drugs to treat the disease as soon as possible; is a result of Iranian cultural belief in overusing drugs, which imposes considerable costs to the insurance industry. In fact, most individuals believe that the more a physician prescribes drugs, the better he is and their evaluation criterion for the selection and referral to physicians is their amount of prescribed drugs. Hafezi et al. stated that the cause of individuals’ dissatisfaction with family physicians and their health team is that those referring to them are rural families and expect them to prescribe many drugs (32). 

Factor affecting the creation of moral hazards from the point of view of participants can be divided into two groups of economic and moral-cultural causes. The false expectations of the insured individuals have roots in individuals’ moral and cultural values. For the insured person, the use of their own health insurance identification cards or that of others is indistinguishable and this issue requires training and powerful supervision as well as cultural construction in the society. Demanding physicians to prescribe drugs and physicians being influenced by patients’ demands in the prescription of drugs are grounds for hazards in the insurance system which should be controlled through training physicians and supervising their prescriptions. Insured individuals should observe the rights of insurance agencies as third party payers and supportive organizations which in cases of diseases can disburden them of the financial load in order that the money obtained from individuals’ participation be spent for individuals’ real needs. The role of religious beliefs should be more highlighted in the society. Moreover, for poor individuals, policies such as special payment reduction or exemption should be implemented to prevent families from facing catastrophic costs and poverty in order to minimize moral hazards in health insurance.
